# The effectiveness of enhanced cognitive behavioural therapy in an outpatient setting

**DOI:** 10.1186/2050-2974-3-S1-O3

**Published:** 2015-11-23

**Authors:** Rachel Signorini, Jeanie Sheffield, Natalie Rhodes, Carmel Fleming, Warren Ward

**Affiliations:** 1 The University of Queensland, St Lucia, QLD, Australia; 2 Eating Disorders Outreach Service, Herston, QLD, Australia

## 

The Eating Disorders Outreach Service (EDOS) at the Royal Brisbane and Women's Hospital has offered Enhanced Cognitive Behaviour Therapy (CBT-E) since 2009. The current research aimed to evaluate the effectiveness of the EDOS CBT-E program by analysing the outcome data of 114 adult outpatients (Mage = 26.06 years, SD = 8.35) with a DSM-IV diagnosis of Anorexia Nervosa, Bulimia Nervosa, or Eating Disorder Not Otherwise Specified. Outpatients attended an average of 20-40 individual sessions with a psychologist or psychiatric registrar. Of those who commenced CBT-E, only 50% completed treatment. Although a higher proportion of non-completers had an Axis II diagnosis, regression analyses revealed that the only significant predictor of drop-out from treatment was the presence of Axis IV psychosocial and environmental problems. Amongst those who completed treatment, CBT-E resulted in statistically and clinically significant improvements in eating disorder and general psychopathology, which were maintained at the 20-week follow-up. When the total sample, including non-completers was considered, statistically (and some clinically) significant improvements in eating disorder and general psychopathology were observed. The findings indicate that CBT-E is an effective treatment for adults with all eating disorders within outpatient settings. However, the high attrition observed indicates that strategies need to be identified to minimise drop-out.

